# National recommendations of the Working group for laboratory diagnostics of autoimmune diseases of the Croatian Society of Medical Biochemistry and Laboratory Medicine: Assessment of antineutrophil cytoplasmic antibodies (ANCA)

**DOI:** 10.11613/BM.2025.020706

**Published:** 2025-06-15

**Authors:** Ana Kozmar, Andrea Tešija Kuna, Nada Tomić Sremec, Lovorka Đerek, Vedrana Drvar, Katarina Gugo

**Affiliations:** 1 Department of Laboratory Diagnostics, University Hospital Centre Zagreb, Zagreb, Croatia; 2 University of Zagreb, Faculty of Pharmacy and Biochemistry, Zagreb, Croatia; 3 Department of Clinical Chemistry, Sestre Milosrdnice University Hospital Center, Zagreb, Croatia; 4 Catholic University of Croatia, Zagreb, Croatia; 5 Clinical Department of Laboratory Diagnostics, Dubrava University Hospital, Zagreb, Croatia; 6 Clinical Department of Laboratory Diagnostics, Clinical Hospital Center Rijeka, Rijeka, Croatia; 7 Medical Laboratory Diagnostic Division, University Hospital of Split, Split, Croatia

**Keywords:** antineutrophil cytoplasmic antibodies, autoimmunity, harmonization, recommendations

## Abstract

The family of antineutrophil cytoplasmic antibodies (ANCA) includes autoantibodies targeting proteins within the primary granules of neutrophils and lysosomes of monocytes. So far, proteinase 3 (PR3) and myeloperoxidase (MPO) are considered clinically relevant ANCA specificities. National recommendations for the assessment of ANCA are the outcome of the survey done by the Working group (WG) for laboratory diagnostics of autoimmune diseases of the Croatian Society of Medical Biochemistry and Laboratory Medicine (CSMBLM), where the diversity in the performance of ANCA testing and reporting among the laboratories in Croatia was observed. This document contains recommendations concerning the indications for ANCA testing, preanalytical, analytical and postanalytical issues, including rational algorithm and quality control assurance. The recommendations are based on the International consensus on ANCA testing and reporting as well as other relevant literature in order to help to harmonize ANCA testing. The aim of these recommendations is to improve and harmonize ANCA testing among laboratories in Croatia.

## Graphical abstract

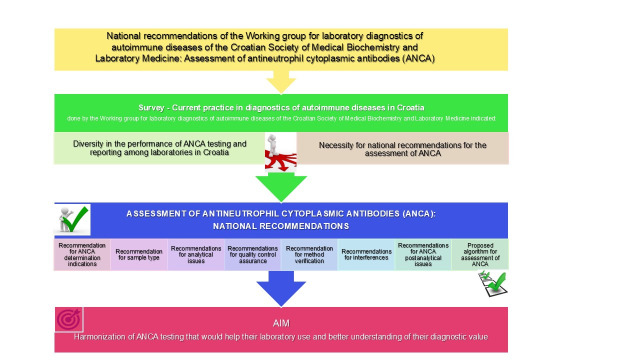

## Highlights

• National recommendations for antineutrophil cytoplasmic antibodies (ANCA) testing based on the International consensuses on ANCA testing and reporting

• Recommendations include indications for ANCA testing, preanalytical, analytical and postanalytical issues, including rational algorithm and quality control assurance

• These recommendations aim to harmonize ANCA testing to enable their laboratory use and better understanding of their diagnostic value

## Introduction

The family of antineutrophil cytoplasmic antibodies (ANCA) encompasses autoantibodies targeting proteins within the primary granules of neutrophils and lysosomes of monocytes. Target candidates include proteins with antibacterial activity such as lysozyme, myeloperoxidase (MPO), or bacterial permeability-increasing protein (BPI) as well as numerous proteases (elastase, proteinase 3 (PR3), cathepsins, *etc.*). However, the only well-known clinically relevant ANCA specificities are PR3 and MPO (*1*).

Historically, indirect immunofluorescence (IIF) on ethanol-fixed human granulocytes has been used for the detection of ANCA. Two major fluorescence patterns are cytoplasmic (C-ANCA) and perinuclear (P-ANCA) and the rest are atypical ANCA. PR3-ANCA is mostly associated with cytoplasmic and MPO-ANCA with perinuclear pattern (*2*, *3*).

Antineutrophil cytoplasmic antibodies targeting MPO or PR3 are a hallmark of the subgroup of small-vessel systemic vasculitis called ANCA-associated vasculitis (AAV) (*1*). ANCA-associated vasculitis is characterized with necrotizing vasculitis predominantly affecting small but also medium-sized vessels, without or with only a few immune deposits (pauci-immune pattern). According to clinicopathological features, 3 subtypes of AAVs are distinguished: granulomatosis with polyangiitis (GPA), microscopic polyangiitis (MPA), and eosinophilic GPA (EGPA). In line with this, the 2012 revised International Chapel Hill Consensus Conference on the Nomenclature of Vasculitides suggested the use of prefix to the clinical phenotype indicating ANCA specificity (PR3-ANCA, MPO-ANCA, or ANCA-negative) (*4*, *5*). Granulomatosis with polyangiitis is mostly associated with PR3-ANCA and MPA with MPO-ANCA (Table 1), while EGPA is ANCA positive in approximately 50% of patients and is more associated with MPO (*1*, *6*).

**Table 1:** Sensitivities and specificities of ANCA-IIF and PR3/MPO immunoassays for diagnosis of GPA and MPA

	**IIF**				**Antigen-specific immunoassays**			
	C-ANCA		P-ANCA		PR3-ANCA		MPO-ANCA	
	Se (%)	Sp(%)	Se (%)	Sp(%)	Se (%)	Sp(%)	Se (%)	Sp(%)
GPA	65-77		11-15		77-81		9-12	
		97-98		81-96		98-99		96-99
MPA	5-6		85-89		5-9		71-88	
Specificity obtained with disease controls. According to Damoiseaux *et al.* (12). IIF - indirect immunofluorescence. ANCA - antineutrophil cytoplasmic antibodies. C-ANCA - cytoplasmic antineutrophil cytoplasmic antibodies. P-ANCA - perinuclear antineutrophil cytoplasmic antibodies. PR3 - proteinase 3. MPO – myeloperoxidase. Se - sensitivity. Sp - specificity. GPA - granulomatosis with polyangiitis. MPA - microscopic polyangiitis.								

Besides the diagnostic role of ANCA in AAV, there is a potential prognostic role since some evidence indicates that PR3-positive AAV is more prone to relapse than MPO-positive AAV (*7*). Antineutrophil cytoplasmic antibodies concentrations often correlate with disease activity and can precede the relapse of the disease so the International consensus on testing and reporting of ANCA suggests serial measurements of previously positive ANCA (*8*). However, the utility in clinical practice is unclear since neither a significant change in antibody concentrations nor measurement intervals are defined (*3*).

Drug-induced vasculitis is another variant of AAV and is related to the use of antithyroid drugs, antibiotics, hydralazine, allopurinol, propylthiouracil, sulfasalazine, minocycline, and anti-tumor necrosis factor-α (TNF-α) inhibitors. It is mostly associated with positive MPO-ANCA in high titres and the coexistence of antinuclear antibodies (*1*).

Antineutrophil cytoplasmic antibodies, especially atypical P-ANCA without well-characterized specificity, are also associated with non-AAV conditions: inflammatory bowel diseases and autoimmune liver diseases. In ulcerative colitis (UC) atypical P-ANCA can be found in 40-70% of patients, while much rarer in Crohn’s disease (CD). Therefore, the detection of ANCA is justified in the differential diagnosis of these two entities.

Atypical P-ANCA is frequently found also in patients with autoimmune liver diseases but lacks specificity due to the presence in liver diseases of viral or alcohol etiology. Determination of ANCA has diagnostic value in autoimmune hepatitis type I in case of the absence of other common autoantibodies (*9*, *10*).

The necessity for national recommendations for the assessment of ANCA came out from the survey launched by the Working group (WG) for laboratory diagnostics of autoimmune diseases of the Croatian Society of Medical Biochemistry and Laboratory Medicine (CSMBLM) (*11*). According to the survey results, ANCA determination is performed in approximately half of the laboratories dealing with the laboratory diagnostics of autoimmune diseases. Most of the laboratories perform only PR3/MPO specific tests while only a minority of labs perform ANCA IIF test and are mostly concordant in all aspects, except for the non-classical ANCA pattern recognition. The most critical finding was the performance of the solid-phase-based ANCA screen assay without specifying the method and target antigens included, which can be misleading in the context of seeking for ANCA in inflammatory bowel disease.

The aim of these recommendations is to improve and harmonize the ANCA testing among the laboratories in Croatia. These recommendations are based on the International consensuses on ANCA testing and reporting in different clinical contexts and also other relevant literature, and cover all critical issues from preanalytical to postanalytical phase: a) indications for ANCA testing; b) sample type and stability; c) analytical issues regarding different methods in use; d) quality control assurance; e) method verification; f) interferences; g) rationale algorithm; h) report of the results and turnaround time (TAT) (*2*, *9*).

## Preanalytical issues

### Indications for ANCA testing

Recommendation for ANCA determination indications:

1. ANCA testing is mandatory for patients with clinical features suggesting AAV.
2. In non-AAV, ANCA testing is mandatory in patients with anti-GBM disease and idiopathic interstitial pneumonia while recommended in autoimmune rheumatic diseases and infectious diseases only in the case of kidney involvement and also in the case of diagnostic uncertainty to discriminate UC from CD.
3. It is not recommended to monitor disease activity in patients with AAV based on ANCA concentrations alone.

Antineutrophil cytoplasmic antibodies are a valuable laboratory marker to support the diagnosis of AAV including GPA, MPA, and EGPA. To determine if ANCA testing is advisable, ANCA testing should be requested for patients with clinical indications shown in Table 2 (*2*).

**Table 2:** Clinical indications for ANCA testing

Glomerulonephritis, especially rapidly progressive glomerulonephritis
Pulmonary haemorrhage, especially pulmonary renal syndrome
Cutaneous vasculitis with systemic features
Multiple lung nodules
Chronic destructive disease of the upper airways
Long-standing sinusitis or otitis
Subglottic tracheal stenosis
Mononeuritis multiplex or other peripheral neuropathy
Retro-orbital mass
Scleritis
Reproduced with permissions from Judy Savige *et al*. (8).

The pre-test probabilities for AAV associated with the clinical presentations improve clinical interpretation of test results. It is recommended to request ANCA testing for patients with high pre-test probability for AAV (*12*).

When monitoring disease activity in AAV, increases in ANCA concentrations are only modestly informative as an indicator of disease activity and are not reliable predictors of disease flares for individual patients, even though some studies showed that up to 57% of relapses were preceded by a rise in ANCA up to 8 weeks earlier (*13*). Increasing immunosuppressive therapy based on changes in ANCA concentrations alone can result in unnecessary immunosuppression resulting in adverse events. Persistence of ANCA positivity does not necessarily indicate that continued immunosuppressive therapy is required (*14*). Despite the current lack of consensus regarding the monitoring of patients by ANCA concentration, it can often be one of the valuable elements in making a clinical decision (*15*).

Antineutrophil cytoplasmic antibodies testing may help in the diagnosis of other diseases beyond AAV. According to the 2020 international consensus on ANCA testing beyond systemic vasculitis in patients with other autoimmune diseases ANCA may have clinical and pathogenic relevance, while in other disorders ANCA testing may be helpful for differential diagnosis or to support diagnosis. For example, all patients with anti-glomerular basement membrane (anti-GBM) disease, idiopathic interstitial pneumonia (IIP) or infective endocarditis associated with nephritis should be tested for ANCA. 2020 international consensus on ANCA testing beyond systemic vasculitis was prepared by a group of 29 experts that voted for each statement using a 5-point Lickert scale. All recommendation statements have reached a high level of agreement (Table 3).

**Table 3:** Recommendations statements for ANCA testing in non-AAV

**Disease**	**Statements**	**LoA, %**	**Median score**
Any disease	ANCA testing* is mandatory for any patient with clinical features suggesting AAV	100	5
Anti-GBM disease	ANCA testing* is mandatory for any patient with anti-GBM disease	100	5
Idiopathic interstitial pneumonia	ANCA testing* is mandatory for any patient with idiopathic interstitial pneumonia	96.6	5
Rheumatoid arthritis	Routine testing is not recommended. Recommended* in patients with kidney disease with a nephritic sediment	96.6	5
Systemic lupus erythematosus	Routine testing is not recommended. Recommended* in patients with a kidney biopsy with prominent necrotizing and crescentic lesions or proliferative lupus nephritis	100	5
Systemic sclerosis	Routine testing is not recommended. Recommended* in patients with kidney disease with a nephritic sediment	96.6	5
Primary Sjögren’s syndrome	Routine testing is not recommended. Recommended* in patients with kidney disease with a nephritic sediment	96.6	5
Autoimmune liver diseases (AIH-1, PBC, PSC)	Routine testing is not recommended. Testing for ANCA by IIF may be useful in patients with suspected AIH-1 in the absence of conventional autoantibodies. Patients with autoimmune liver disease usually develop atypical P-ANCA.	89.7	5
Inflammatory bowel diseases (CD, UC)	Routine testing is not recommended. In case of diagnostic uncertainty to discriminate UC from CD atypical P-ANCA (IIF) and ASCA may be tested	96.6	5
Infections	Routine testing is not recommended. In patients with renal impairment, especially associated with infective endocarditis ANCA testing may be useful.	89.7	5
Malignancy	Routine testing is not recommended	96.6	5
*MPO-ANCA and PR3-ANCA according to the 2017 consensus. LoA - level of agreement (%). Scores: 1 - strongly disagree, 2 - disagree, 3 - undecided, 4 - agree, 5 - strongly agree. Reproduced with permissions from Moiseev *et al.* (9). AIH-1 - autoimmune hepatitis type 1. PBC - primary biliary cholangitis. PSC - primary sclerosing cholangitis. CD - Crohn’s disease. UC - ulcerative colitis. ASCA - anti-*Saccharomyces cerevisiae* antibodies. ANCA - antineutrophil cytoplasmic antibodies. P-ANCA - perinuclear antineutrophil cytoplasmic antibodies.			

## Sample type and stability

Recommendation for sample type:

1. The recommended sample type for ANCA determination is serum.

The specimen of choice for ANCA testing is serum (serum gel tubes have not been tested for possible interference). Samples can be stored at 4 °C for two days and for longer periods they should be stored at - 20 °C or lower. Frozen specimens must be mixed well after thawing and multiple freeze/thaw cycles which may cause loss of antibody activity should be avoided. Some manufacturers of commercial tests for detection of ANCA (IIF), MPO or PR3 recommend serum and plasma as acceptable samples. According to most manufacturers, grossly hemolyzed, lipemic or icteric samples should not be tested outside of the interfering concentration specified by the manufacturer or established by own study (*16*).

## Analytical issues

Recommendations for analytical issues (general):

1. The primary screening method for ANCA in AAV should be a high-quality antigen-specific immunoassay for PR3-ANCA and MPO-ANCA.
2. In cases of negative findings for PR3-ANCA and MPO-ANCA, but with a significant clinical indication for AAV, it is recommended to use IIF method on ethanol-fixed granulocytes (preferable in combination with formalin-fixed granulocytes) and in the case of negative result consider use of alternative high-quality antigen-specific immunoassay. The same recommendation applies in cases of a weakly positive test result in order to increase specificity.
3. In case of an unknown referral diagnosis, it is recommended to use indirect immunofluorescence on ethanol-fixed granulocytes (preferably in combination with formalin-fixed granulocytes) as a screening method. In patients with positive ANCA or any cytoplasmic fluorescence, it is necessary to determine antibodies to MPO and PR3 using high-quality immunoassays.
4. If IIF is applied as a primary screening method for ANCA, the laboratory is obliged to ensure that the IIF operates at a high level of sensitivity.
5. The conjugate used for the detection of ANCA should be specific for the IgG isotype of antibodies.

According to the 1999 International Consensus Statement on Testing and Reporting of Antineutrophil Cytoplasmic Antibodies, the recommended screening method for ANCA detection was indirect immunofluorescence (IIF) on ethanol-fixed human neutrophils. Positive results were always followed by specific testing for PR3 and MPO antibodies (*8*).

Based on the results of the 2016 multicentre study by the European Vasculitis Study Group (EUVAS) conducted by Damoiseaux *et al.*, revised international consensus on testing of ANCA in GPA and MPA was adopted in 2017 (*6*). According to the new consensus statement, in case of suspicion of AAV, high-quality immunoassays for MPO-ANCA and PR3-ANCA should be used as primary screening methods. Most third-generation PR3-ANCA and MPO-ANCA assays can be considered as high-quality assays since they demonstrate equal or better diagnostic performance than IIF for AAV diagnosis (*17*). High-quality immunoassays showed greater specificity and sensitivity for newly diagnosed GPA and MPA compared to IIF. The new consensus therefore emphasizes that IIF should not be used as a primary screening method in cases of high probability for AAV. Indirect immunofluorescence or alternative high-quality immunoassays are used in cases of weakly positive results of the primary screening immunoassay or in cases of negative results for MPO-ANCA or PR3-ANCA when there is a high clinical suspicion for the diagnosis of AAV (*2*).

Although IgA and IgM antibodies can be present, the conjugate used for the detection of ANCA by the IIF method as well as for specific MPO and PR3 antibodies should be specific for the IgG isotype as only these antibodies have proven clinical significance.

## Myeloperoxidase-ANCA and PR-3-ANCA specific immunoassays in ANCA testing

Recommendations for analytical issues (solid phase assay):

1. It is recommended to use quantitative tests for PR3-ANCA and MPO-ANCA detection because antibody concentration indicates the probability of AAV.
2. Use of qualitative methods for MPO/PR3 specificity should be limited to rapid ANCA detection or only in conjunction with ANCA IIF method.

In the last twenty years, there have been significant advances in the diagnostic characteristics of tests for ANCA detection. First-generation immunoassays (antigen attached directly to the plate) were replaced by second-generation tests (antigen attached to a monoclonal antibody) and third-generation tests (antigen attached to a peptide carrier), which increased sensitivity. Various immunochemical tests are available now for the detection of PR3 and MPO antibodies, for example, ELISA (enzyme-linked immunosorbent assay), FEIA (fluorescence enzyme immunoassay), CLIA (chemiluminescent immunoassay), ALBIA (addressable laser bead immunoassay) and LIA (line immunoassay) (*2*, *18*, *19*).

Depending on the clinical context, quantitative and qualitative tests can be used to determine PR3 and MPO antibodies. For routine screening for diagnostic purposes, it is recommended to use quantitative immunochemical tests for PR3-ANCA and MPO-ANCA measurement because the information on the antibody concentration indicates the probability of ANCA-vasculitis (*12*). Qualitative immunochemical tests can be used as an alternative confirmatory method and in cases when rapid determination of ANCA antibodies is needed. Pulmonary-renal syndrome is a condition that requires rapid testing for ANCA and GBM antibodies. In this setting, it is recommended to use tests that simultaneously detect GBM antibodies in addition to PR3 and MPO antibodies. The newly positive results of GBM antibodies represent a critical result and should be communicated directly to the clinician. The same applies to ANCA results when rapid testing is ordered. Positive results of qualitative tests should be confirmed with a quantitative test (*20*).

Despite the lack of standardization, the diagnostic value of ANCA testing is increased through the harmonization of antibodies testing. Laboratories are encouraged to apply the recommended screening algorithm for ANCA antibodies using adequate high quality immunoassays. Also, rational test ordering based on gating strategies established on clinical manifestations associated with AAV contributes to increase of diagnostic specificity (*2*, *21*).

## Indirect immunofluorescence in ANCA testing

Recommendations for analytical issues (indirect immunofluorescence testing):

1. Three main patterns of fluorescence should be identified using ethanol-fixed human granulocytes: C-ANCA or cytoplasmic pattern, P-ANCA or perinuclear pattern and atypical ANCA.
2. In the case of ANA immunofluorescence pattern that is difficult to separate from the ANCA pattern, the use of Hep-2 cells together with ethanol and formalin-fixed granulocytes is recommended in order to avoid the possibility of misinterpretation of ANCA. But if the presence of ANCA cannot be obtained with certainty this should be stated in the report.
3. Samples found positive on IIF should be diluted until the fluorescence is still visible and the end point titre should be reported.

For detection of ANCA using the IIF method, ethanol-fixed human granulocytes are used as a substrate. There are three main patterns of fluorescence identified using ethanol-fixed human granulocytes (*20*, *22*, *23*). The use of additional substrates including ethanol and formalin-fixed granulocytes together with Hep-2 cells is highly encouraged to avoid misinterpretation of ANCA.

### C-ANCA or cytoplasmic pattern

Cytoplasmic pattern is characterized by a fine granular fluorescence of the cytoplasm, with central, interlobular accentuation. The main target antigen is PR3, serine protease found in azurophil (primary) granules of neutrophil granulocytes and monocytes. Other target antigens associated with this fluorescence pattern are BPI and much less frequently MPO.

### P-ANCA or perinuclear pattern

Perinuclear pattern is characterized by strong fluorescence of the periphery of the nucleus with weak fluorescence of the central part of the nucleus. Such fluorescence pattern is caused by an artefact of granulocytes fixation on ethanol that leads to the migration of positively charged granule components towards the negatively charged nuclear membrane. Clinically the most significant antigen associated with the P-ANCA fluorescence pattern is MPO. Other target antigens associated with P-ANCA include lysozyme, cathepsin G, azurocidin, elastase, lactoferrin, actin, BPI and rarely PR3. In some cases, distinguishing a true positive P-ANCA from ANA antibodies is possible only by using formalin as a fixative. On formalin, true positive P-ANCA will give a cytoplasmic fluorescence pattern, while ANA will be negative. When formalin is used as a fixative of human granulocytes, it prevents the migration of positively charged antigens towards the perinuclear zone. That is the reason why P-ANCA pattern seen on formalin-fixed granulocytes has C-ANCA pattern. As previously mentioned, the use of formalin is important for distinguishing true positive P-ANCA from positive ANA. C-ANCA shows similar granular cytoplasmic fluorescence on ethanol and formalin-fixed granulocytes.

### Atypical ANCA (a-ANCA)

Atypical ANCA is characterized by a fluorescence pattern that may have some characteristics of C-ANCA, P-ANCA or ANA. It is associated with conditions such as RA, connective tissue diseases, autoimmune liver diseases, inflammatory bowel diseases, infections and drugs induced autoimmunity.

### Atypical C-ANCA (a/C-ANCA)

Atypical C-ANCA is characterized by diffuse intense cytoplasmic fluorescence without interlobular accentuation.

### Atypical P-ANCA (a/P-ANCA)

Atypical P-ANCA is characterized by an intense fluorescence of perinuclear zone.

### Atypical inconclusive ANCA (a/X-ANCA)

Any other fluorescence pattern not previously described.

In case of multiple positive immunofluorescence, each type of immunofluorescence should be described.

**Figure 1:**
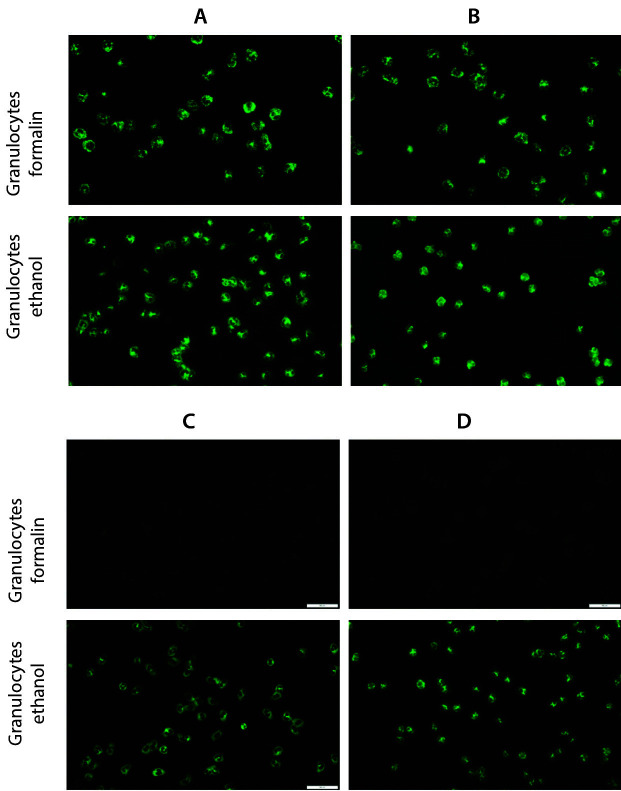
ANCA fluorescence pattern on ethanol (bottom row) and formalin (upper row)- fixed human granulocytes. A) C-ANCA; B) P-ANCA; C) atypical C-ANCA and D) atypical P-ANCA. ANCA - antineutrophil cytoplasmic antibodies. C-ANCA - cytoplasmic antineutrophil cytoplasmic antibodies. P-ANCA - perinuclear antineutrophil cytoplasmic antibodies.

ANCA fluorescence patterns on ethanol and formalin-fixed human granulocytes are shown in Figure 1.

**Figure 2:**
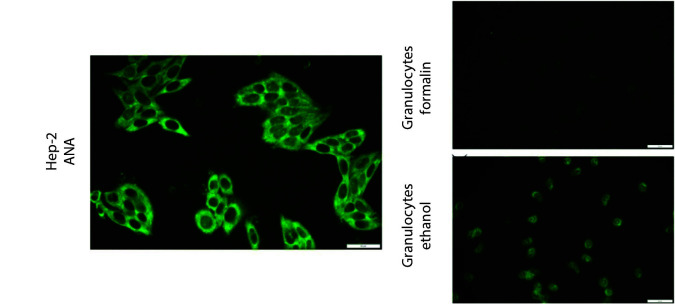
Positive ANA on Hep-2 cells (cytoplasmic fluorescence pattern) that gives atypical cytoplasmic fluorescence on ethanol-fixed granulocytes and no fluorescence on formalin-fixed granulocytes. ANA - antinuclear antibodies.

In case of ANA immunofluorescence pattern that is difficult to separate from the ANCA pattern, the use of Hep-2 cells together with ethanol and formalin-fixed granulocytes is recommended in order to avoid the possibility of misinterpretation of ANCA. If the presence of ANCA cannot be obtained with certainty, this should be stated in the report. An example of a positive ANA on Hep-2 cells (cytoplasmic fluorescence pattern) together with ethanol and formalin-fixed granulocytes is shown in Figure 2.

Pictures from Figure 1 and 2 were taken using Olympus BX43 fluorescent microscope (Olympus, Tokyo, Japan) at the Department of Clinical Chemistry, Sestre milosrdnice University Hospital Center, Zagreb, Croatia. This microscope was purchased through the grant KK.01.1.1.02-0014 of the European Regional Development Fund.

Samples found positive on IIF should be diluted until the fluorescence is still visible, and the end point titre should be reported.

The reagent manufacturer should recommend optimal sample dilution used for ANCA screening (*e.g.* 1:10, 1:20). However, since different factors can influence optimal screening titre, if applicable, laboratories are encouraged to perform the verification of screening titre. For this purpose, sufficient samples with adequate clinical information should be collected through long-term collection and storage or by providing enough samples in cooperation with other laboratories. Confirmation of the cut-off value for the qualitative method and reference interval for the quantitative method should be performed according to available recommendations (*24*, *25*).

## Quality control

Recommendations for quality control assurance:

1. The minimum requirement for a quality control sample is to use the quality controls provided by the manufacturer. The values obtained must fall within the interval specified by the manufacturer to meet the criteria for reporting patient sample results. However, it is highly recommended to use kit independent commercial controls or previously analysed native patient samples as control samples for internal quality control (IQC).
2. When using a kit-independent control (commercial or native patient sample) it is desirable to use control samples which could be analyzed in the same manner as patient samples. In the case of PR3 and MPO specific assays, it is recommended to use control samples with the target values near the defined cut-off values.
3. Internal quality control analysis should be adapted to the type of the test and the dynamic of testing:
  - For IIF assays, it is recommended to use a positive control for each fluorescence pattern and a negative control at least once per lot. In each batch of IIF analysis, at least one positive and negative control samples should be analyzed. It is also recommended to use a native patient serum sample with a known titre as a control for defining the ANCA type as well as the titre itself.
  - For solid-phase-based assays which are performed in batches, IQC should be performed once per batch of patient samples. For solid-phase-based assays which are performed on a daily basis, IQC should be performed every day before the start of the measurement procedure. If previously analyzed patient samples are used for IQC, it is important to define the acceptance criteria, which can be defined as qualitative concordance with the previously obtained results or acceptable quantitative deviation taking into account the coefficient of variation (CV) value defined for the respective method. The frequency of IQC can be modified after a risk analysis has been performed.
4. Participation in external quality control (EQC) schemes is mandatory.

For quality control of ANCA assays the same rules apply as for other autoantibody assays. The quality control rules for ANCA methods are implemented in the accreditation guidelines that apply to all autoantibody detection methods (*20*, *21*, *26*). This includes control of the reagents quality, internal quality control (IQC) and external quality control (EQC).

Reagent quality testing is mandatory for each new lot of reagents. According to the manufacturer’s recommendations, the analysis of the negative and positive samples provided by the manufacturer in each reagent package should be conducted in order to meet the minimum quality control requirement. Line blot and multiplex immunoassays as ALBIA contain internal control of each assay run, but analysis of separate IQC samples is recommended. For all ANCA assays it is highly recommended to additionally perform the analysis of manufacturer-independent commercial controls or native patient samples with target values near the cut-off value since using only kit-dependent controls is not informative enough. Using kit independent previously analyzed control samples (commercial controls or native patient samples) is also recommended in order to detect lot-to-lot variations of reagents. For this purpose, using control samples close to the clinical decision levels is also advisable. This helps detect variations in reagents that could potentially affect clinical decision-making. When possible, it is desirable to analyze control samples (commercial controls or in-house control samples) in the same manner as patient samples. Analyzing control samples in the same manner as patient samples allows for more comprehensive control over all steps of the analytical process.

For IIF assays it is recommended to use a positive control for each classical fluorescence pattern and negative control at least once per lot. In each batch of IIF analysis, at least one positive and negative control sample should be analyzed (*27*). Ideally, analyzing the IQC sample on every slide in the run would be the optimal approach to ensure the detection of interference of background staining or errors in the analytical process (*e.g*. error in the conjugate addition step). However, this approach is hardly applicable in real life, especially in high throughput laboratories, since it makes a significant financial burden and yet does not exclude all possible errors. Analyzing a native patient serum sample with previously established ANCA results can serve as a control for determining both the ANCA titre and the fluorescence pattern.

For solid-phase-based assays which are performed in batches, IQC should be performed once per batch of patient samples. For solid-phase-based assays, which are performed on a daily basis, IQC should be performed once per day before starting the measurement procedure for patient samples. However, different IQC frequencies can be adopted after performing the risk analysis (*28*). If previously analyzed patient samples are used for IQC, it is important to define the acceptance criteria, which can be defined as qualitative concordance with the previously obtained results or acceptable quantitative deviation taking into account the CV value defined for the respective method. According to good laboratory practice, the results of quality control analysis should be adequately documented and analyzed according to predefined acceptance criteria. In the case of unacceptable IQC results, corrective actions should be performed.

Additional information on proficiency of ANCA testing in certain laboratory can be obtained from a retrospective analysis of the proportion of positive samples in a certain time period, but this could be influenced by different factors (*20*).

Participation in EQC is mandatory for all ANCA assays in order to follow-up assay performance.

## Method verification

Recommendation for method verification:

1. Before implementation of a new method for ANCA detection it is necessary to perform analytical verification of the assay.

Implementation of new immunoassays for ANCA detection in laboratory practice requires analytical verification to be performed. Since all requirements of accreditation documents (ISO 15189) are not applicable in this setting, guidelines formulated for specific requests for autoimmunity testing should be consulted (*26*). There are no specific recommendations for method verification in ANCA testing. Each laboratory should choose appropriate existing guidelines for method verification to ensure that test performance meets predefined specifications.

Measurement of assay precision (intra- and inter-assay reproducibility) is an essential part of verification process. Results from EASI study reported intra-assay CV about 10% and inter-assay CV about 15% for majority laboratories for both MPO and PR3 antibodies (*29*).

## Interferences

Recommendations for interferences:

1. Since positive ANA can interfere with ANCA IIF testing, it is crucial to use formalin-fixed granulocytes and HEp-2 cells in addition to ethanol-fixed human granulocytes to prevent misinterpretation of ANCA IIF results in cases of positive ANA.
2. Possible interference in the assay should be suspected in cases of discrepancy in the results obtained with two different immunoassays (for example IIF and solid-phase-based immunoassay) or in case of results that are not in accordance with the clinical condition of the patient.
3. In case of suspected interference in ANCA measurement, testing with an alternative immunoassay should be performed.
4. Performing serial dilution of a patient sample could be done in order to detect possible interferences in ANCA testing (*e.g.* hook effect).

In addition to the interferences of non-specific preanalytical variables, such as hemolysis and/or lipemia, in immunoassays the largest share of interferences is analyte-dependent. There are several different factors that lead to development of the interference in immunoassays as the presence of autoantibodies (for example, the presence of anti-nuclear antibodies (ANA) in the determination of ANCA on ethanol-fixed granulocytes by the IIF method), heterophile antibodies (HA) and human anti-animal antibodies (HAAA) (*28*, *30*, *31*).

When determining ANCA by the IIF method on ethanol-fixed granulocytes, a false positive finding of P-ANCA pattern is possible in the case of positive ANA (especially in the case of positive antibodies to anti-dsDNA) because of the reorganization of nuclear proteins, which causes a fluorescence pattern similar to P-ANCA pattern. When IIF and immunoassay are simultaneously used possible interference should be suspected in cases of discrepancy in the obtained results or in the case of results that are not in accordance with the clinical condition of the patient (*28*, *31*). For example, highly positive result of PR3 or MPO immunoassay with negative or borderline fluorescence on ethanol-fixed granulocytes, the prozone effect in IIF method should be suspected in such case.

## Rational algorithm

The appropriate sequence of methods used for assessment of ANCA depends on the clinical context. In the presence of clear clinical suspicion of certain diagnoses (GPA and MPA), adequately referred to the laboratory, the algorithm should stem from the current international recommendations for laboratory diagnostics of those conditions (*2*). These recommendations suggest omitting the screening IIF test in favor of immediately testing for presence of anti-MPO and anti-PR3 antibodies using a high quality assay. In the case of positive result of specific autoantibodies, there is no need for further testing. However, in case of negative or borderline results, an IIF test or an alternative solid-phase-based assay should be carried out. Similar algorithm is adequate for another entity - EGPA (*9*).

**Figure 3:**
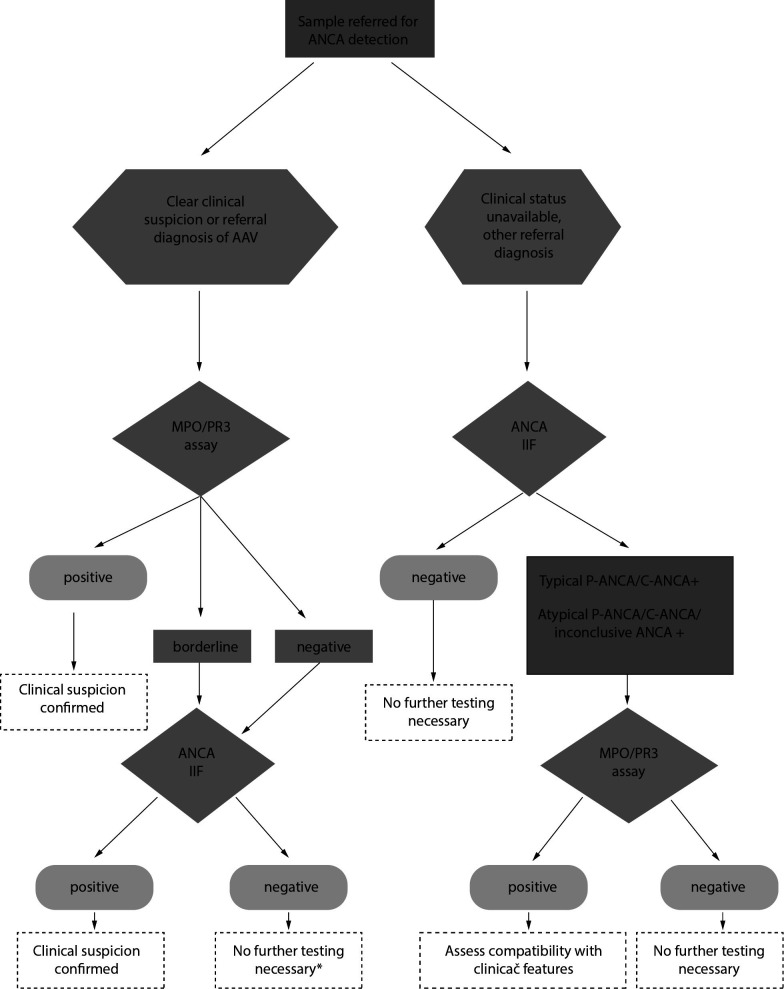
Proposed algorithm for assessment of ANCA. *Consider alternative high-quality test in case of a high clinical suspicion. IIF - indirect immunofluorescence. C-ANCA - cytoplasmic antineutrophil cytoplasmic antibodies. P-ANCA - perinuclear antineutrophil cytoplasmic antibodies. PR3 - proteinase 3. MPO - myeloperoxidase. AAV - ANCA-associated vasculitis. ANCA - antineutrophil cytoplasmic antibodies.

A different approach is necessary in the situation, which is more common in everyday practice, when data on patient’s clinical characteristics are scarce or there is an alternative or ambiguous diagnosis in the referral documentation. In this case, it is advisable to adhere to older guidelines (*8*). This international consensus covers assessing of ANCA regardless of the clinical context and postulates the IIF test as the mandatory entry method for all previously untested patients. Samples containing not only classical ANCA, but any cytoplasmic or homogeneous/peripheral nuclear fluorescence should then be tested for presence of anti-MPO and anti-PR3 antibodies using a high-quality assay (*8*). A rational algorithm that encompasses both situations regarding the clinical status is given in Figure 3.

Laboratories unable to perform both testing methods should clearly indicate the limitations of used methodology on the report.

## Postanalytical issues

Recommendations for postanalytical issues:

1. Units of measurement should be reported, for classical ANCA pattern (P-ANCA and C-ANCA) as IIF titre and IU/ml for anti-MPO and anti-PR3.
2. If possible, reporting results based on multiple cut-off values that identify negative, low positive, medium positive and high positive results should be implemented.
3. The method of determination should be stated on the laboratory report.
4. The methods of determination should enable anti-MPO and anti-PR3 results within 24 hours. Critical results should be reported directly to the clinician.

Several key points require attention in reporting of the results: 1) units of measurement, 2) cut-off, 3) method in use, and 4) turnaround time (TAT).

Depending on the method in use, units of measurement should be reported, especially if solid-phase-based assay is used. Considering that certified reference materials against PR3 IgG and MPO IgG have been developed, it is possible to improve the comparability of results using a commutable certified reference material (CRM) for calibration even though it does not guarantee similar results (*32*, *33*). If so, units of measurements should be IU/mL for anti-MPO and anti-PR3 (*34*, *35*). If IIF method is used the titre should be clearly stated. For proper interpretation of test results, appropriately defined reference ranges for antibody concentrations is mandatory. Usually, for qualitative tests a single cut-off value is used that defines the result as positive or negative. Some manufacturers provide also the equivocal range. Higher ANCA concentrations are associated with higher likelihood ratios (LR), meaning increased certainty of the right disease diagnosis. Considering that, if possible, reporting results based on multiple cut-off values that identify negative, low positive, medium positive and high positive results can facilitate interpretation of the results in clinical context (*12*, *36*, *37*). Laboratories are encouraged to report LR in addition to the ANCA result thus improving clinical interpretation (*38*, *39*).

It is of great importance to clearly state the method in use considering a great number of commercial ANCA assays have become available. Next to the ELISA methods, novel solid-phase-based technologies, like ALBIA, CLIA, CMIA, FEIA, line or dot immunoassays, as well as IIF are in use. Most of the methods have been clinically evaluated but due to the noticed differences the method in use should always be stated.

Considering ANCA testing can be used in different clinical settings, there are situations that require rapid diagnosis like renal-pulmonary syndrome that require result within 24 hours. Therefore, the methods of determination should enable anti-MPO and anti-PR3 results within 24 hours (automated single test methods) and critical results should be reported directly to the clinician (*2*, *6*, *40*, *41*).

## Conclusions

Recently many improvements have been made in the overall quality of ANCA results, especially in relation to antigen-specific immunoassays, which prompted a change in ANCA testing. That encouraged the introduction of the 2017 revised international consensus on ANCA testing in AAV and also the 2020 international consensus on ANCA testing beyond systemic vasculitis. In view of these changes, there was a need to present recommendations for ANCA testing on the national level, taking into account all the specificities of laboratory diagnostics in Croatia. These recommendations aim to harmonize ANCA testing that would improve their laboratory use and better understanding of their diagnostic value.

## Data Availability

No data was generated during this study, so data sharing statement is not applicable to this article.
